# A White Matter Connection of Schizophrenia and Alzheimer’s Disease

**DOI:** 10.1093/schbul/sbaa078

**Published:** 2020-07-18

**Authors:** Peter Kochunov, Artemis Zavaliangos-Petropulu, Neda Jahanshad, Paul M Thompson, Meghann C Ryan, Joshua Chiappelli, Shuo Chen, Xiaoming Du, Kathryn Hatch, Bhim Adhikari, Hemalatha Sampath, Stephanie Hare, Mark Kvarta, Eric Goldwaser, Fude Yang, Rene L Olvera, Peter T Fox, Joanne E Curran, John Blangero, David C Glahn, Yunlong Tan, L Elliot Hong

**Affiliations:** 1 Department of Psychiatry, Maryland Psychiatric Research Center, University of Maryland School of Medicine, Baltimore, MD; 2 Imaging Genetics Center, Mark & Mary Stevens Neuroimaging & Informatics Institute, Keck School of Medicine, University of Southern California of USC, Marina del Rey, CA; 3 Beijing Huilongguan Hospital, Peking University Huilongguan Clinical Medical School, Beijing, P. R. China; 4 Department of Psychiatry, University of Texas Health Science Center at San Antonio, San Antonio, TX; 5 Research Imaging Institute, University of Texas Health Science Center at San Antonio, San Antonio, TX; 6 Department of Human Genetics and South Texas Diabetes and Obesity Institute, School of Medicine, University of Texas Rio Grande Valley, Brownsville, TX; 7 Department of Psychiatry, Boston Children’s Hospital, Harvard Medical School, Boston, MA

**Keywords:** white matter deficit pattern, schizophrenia, Alzheimer’s disease, dementia

## Abstract

Schizophrenia (SZ) is a severe psychiatric illness associated with an elevated risk for developing Alzheimer’s disease (AD). Both SZ and AD have white matter abnormalities and cognitive deficits as core disease features. We hypothesized that aging in SZ patients may be associated with the development of cerebral white matter deficit patterns similar to those observed in AD. We identified and replicated aging-related increases in the similarity between white matter deficit patterns in patients with SZ and AD. The white matter “regional vulnerability index” (RVI) for AD was significantly higher in SZ patients compared with healthy controls in both the independent discovery (Cohen’s *d* = 0.44, *P* = 1·10^–5^, *N* = 173 patients/230 control) and replication (Cohen’s *d* = 0.78, *P* = 9·10^–7^, *N* = 122 patients/64 controls) samples. The degree of overlap with the AD deficit pattern was significantly correlated with age in patients (*r* = .21 and .29, *P* < .01 in discovery and replication cohorts, respectively) but not in controls. Elevated RVI-AD was significantly associated with cognitive measures in both SZ and AD. Disease and cognitive specificities were also tested in patients with mild cognitive impairment and showed intermediate overlap. SZ and AD have diverse etiologies and clinical courses; our findings suggest that white matter deficits may represent a key intersecting point for these 2 otherwise distinct diseases. Identifying mechanisms underlying this white matter deficit pattern may yield preventative and treatment targets for cognitive deficits in both SZ and AD patients.

## Introduction

Compared with the general population, individuals with schizophrenia (SZ) have a significantly higher risk (2–4 times) of developing Alzheimer’s disease (AD) and other dementias.^1^ As the hallmark neuropathological features of AD are not typically found in younger SZ patients,^2,3^ this elevated risk for AD remains unexplained. Literature on this topic inevitably invokes Kraepelin’s one-hundred-year-old term *dementia praecox,* describing the intellectual decline and observed neurodegeneration in some SZ postmortem examinations.^4^ However, the historical definition of SZ^5,6^ and the modern epidemiological literature^1,7^ do not offer a mechanistic explanation for the higher rate of AD development in SZ patients.

Patients with AD and SZ both express complex anatomical and cognitive deficits but with nonoverlapping clinical presentations. AD is characterized by a relatively rapid progression with illness onset occurring during the sixth to eighth decades of life. In contrast, SZ typically emerges between late adolescence and early adulthood with rapid worsening of cognitive deficits following illness onset and a gradual aging-related decline afterward.^8–10^ The dominating etiological pathways for SZ and AD are likewise nonoverlapping. The prevailing hypothesis in AD posits neuronal atrophy due to the accumulation of β-amyloid (Aβ) plaques and τ-neurofibrillary tangles. The prevailing hypotheses in SZ are dysfunctions in the dopamine, glutamate, and other neurotransmitter circuits.^11–13^ AD and SZ are distinct in their clinical presentations; however, we may have overlooked an important shared feature between AD and SZ such as the profound white matter impairment in both illnesses.

Despite etiological and clinical differences, impaired brain white matter microstructure, measured as a reduction in fractional anisotropy (FA) derived from diffusion tensor imaging (DTI), is prominent in both disorders: white matter deficits are present before disease onset and track with the rise in cognitive decline and symptom severity.^10,14–16^ In AD, the illness-related pathology is characterized by distinct temporal and regional patterns that track with the accumulation of Aβ plaques and τ-neurofibrillary tangles.^17^ AD pathology is considered a direct factor in axonal damage and the regional specificity of FA deficits, independent of age and hyperintensive white matter lesion burden.^18^ Reduced FA values in pathways serving higher cognitive function are associated with higher cognitive deficits in AD.^16^ In SZ, regional FA deficits also occur in specific temporal and regional patterns that precede symptoms and are associated with cognitive deficits.^19–30^ A large and inclusive meta-analytic SZ study performed by the Enhancing Neuro Imaging Genetics Meta-Analysis (ENIGMA) consortium showed that patients have reproducible and stable patterns of white matter deficits.^31^ Here, we aimed to evaluate the similarity of the white matter deficit patterns between AD and SZ as a potential explanation for the higher risk of developing AD in SZ patients.

To capture the similarity between white matter deficit patterns, we calculated a white matter regional vulnerability index (RVI)^32^ for AD using data from the Alzheimer’s Disease Neuroimaging Initiative (ADNI). RVI quantifies the *similarity* between white matter patterns of any individual and the deficit pattern observed in AD. We tested the hypothesis that SZ patients have elevated RVI scores for AD in independent discovery, replication, and meta-analytical samples. We tested specificity by contrasting deficit patterns in AD with patterns in mild cognitive impairment (MCI). MCI has elevated risks for developing AD but itself is not AD; thus, we used the similarity to MCI as a control for testing age-related anatomical deficits that are AD specific. We further tested a reverse association by showing elevated SZ white matter deficit patterns in AD patients. Finally, we tested the hypothesis that RVI-defined white matter patterns impact the cognitive deficits most characteristic of SZ and AD.

## Materials and Methods

### Sample Characteristics

The AD and MCI deficit patterns were derived from datasets collected and distributed by the ADNI. We chose 3 independent SZ samples: a sample of patients and controls from the Baltimore, MD area to serve as the discovery sample; a sample of patients and controls from Beijing, China, to serve as the replication sample; and a meta-analytical sample assessed by ENIGMA-SZ workgroup to serve as another replication sample. The ethnic and geographical diversity of these samples was meant to reduce cohort-specific variances in diagnosis, treatment, and other factors. All DTI data were processed using a consistent ENIGMA-DTI workflow^33^ and passed comprehensive QA and QC steps. Detailed demographic (supplementary tables S1 and S2) and imaging information (supplementary table S3) are in the supplementary information. Briefly:

#### ADNI Samples: AD and MCI

We downloaded demographic, diagnostic, and imaging data for participants in ADNI-2 protocols (http://adni.loni.usc.edu). The details of the site and imaging protocols are provided in the SI. The DTI data passed ENIGMA QA/QC steps for 218 participants. This sample included 53 healthy controls (M/F = 24/29, age = 72.77 ± 7.57), 117 MCI (M/F = 73/44, age = 72.70 ± 7.67), and 48 AD (M/F = 29/19, age = 72.57 ± 7.67) patients. The cognitive data included: Mini Mental Status Examination (MMSE),^34^ Clinical Dementia Rating Scale Sum-of-Boxes (CDR-SOB),^35^ and Alzheimer’s Disease Assessment Scale-Cognitive subscale (ADAS-Cog).^36^

#### Discovery Sample

The sample consisted of 173 SZ patients (M/F = 122/51, age = 36.0 ± 12.7) and 210 controls (M/F = 119/111, age = 38.2 ± 13.0) from the Greater Baltimore area for whom DTI data passed ENIGMA QA/QC requirements. Patients were recruited from community psychiatric clinics and diagnosed with either DSM-IV schizophrenia or schizoaffective disorder. Controls had no Axis I psychiatric disorder. Cognition was evaluated by the Digit Symbol Coding task of the WAIS-3^37^ to assess processing speed and the Digit Sequencing Test^38^ to assess working memory (supplementary table S2). The clinical and DTI protocol information have been reported^9^ and are further described in SI.

#### Replication Sample

The sample consisted of 122 SZ patients (M/F = 57/65, 38.2 ± 13.3) and 78 controls (M/F = 37/41, 39.2 ± 14.0), recruited from Beijing Huilongguan Hospital, Beijing, China for whom DTI data passed ENIGMA QA/QC requirements. Controls were recruited through local advertisements. Patients met DSM-IV criteria for SZ, and all participants were Han Chinese. DTI data were collected using a 3T Siemens MRI scanner. The clinical and DTI protocol information have been reported^32^ and are further described in SI.

#### Meta-Data Replication Sample From ENIGMA

The worldwide aggregate data from the largest meta-analysis of DTI in SZ performed by ENIGMA^39^ were used as the second replication sample. This sample consisted of 1963 SZ patients and 2359 controls from 29 international studies.^39^ The clinical, DTI, and harmonization information have been described previously.^39^

### Image Processing

**Fig. 1.**
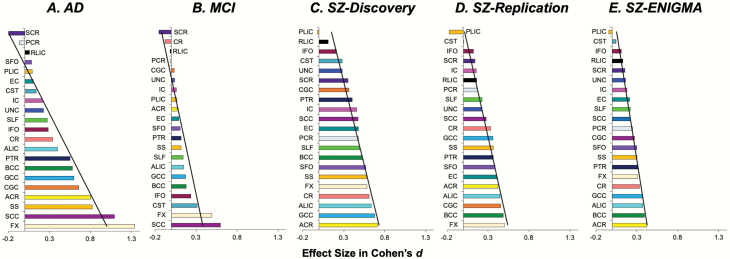
The pattern of white matter microstructural deficits across 22 white matter regions as ranked by their Cohen’s *d* effect sizes (patients < controls) in fractional anisotropy in each sample. The tabulated statistics are presented in supplementary table S5. The black line indicates the best linear fit for each disease; regional vulnerability index (RVI) (eg, for AD) is essentially a calculation of the correlation coefficient between this line for AD and the FA of these 22 regions in an individual. *Note*: ACR, Anterior Corona Radiata; ALIC, Anterior Limb of Internal Capsule; BCC, Body of Corpus Callosum; CC, Corpus Callosum; CGC, Cingulum; CR, Corona Radiata; CST, Cortico-Spinal Tract; EC, External Capsule; FX, Fornix, GCC, Genu of Corpus Callosum, IC, Internal Capsule; IFO, Inferior Frontal Occipital fasciculus; PCR, Posterior Corona Radiata; PLIC, Posterior Limb of Internal Capsule; PTR, Posterior Thalamic Radiation; RLIC, Retrolenticular Limb of the Internal Capsule; SCC, Splenium of Corpus Callosum; SCR, Superior Corona Radiata; SFO, Superior Fronto-Occipital Fasciculus; SLF, Superior Longitudinal Fasciculus; SS, Sagittal Striatum; UNC, Uncinate Fasciculus.

DTI data for all cohorts were processed uniformly using the ENIGMA-DTI analysis pipeline (https://www.nitrc.org/projects/enigma_dti)^33^; details are described in SI. The per-tract effect size for the FA, shown in figure 1 (see legend for tract names), was calculated by averaging values along tract regions of interest in both hemispheres. All data were corrected for age, sex, and age by sex interaction during normalization to increase compatibility among the cohorts.

### Statistical Analysis

#### RVI Calculation

The RVI is a simple measure of the similarity between an individual and the expected deficit pattern derived from a representative disease sample.^31,32^ In this study, it is calculated as a correlation between an individual’s pattern of FA in the 22 white matter regions and the expected pattern of a disease condition. Regional FA for each tract was *z*-normalized by calculating the residual values after the regression of age and sex effects, and then subtracting the average value for a region and dividing it by the standard deviation calculated from the sample’s healthy controls. This produced a vector of *z*-values with 22 regional values for every individual. The ADNI sample was used to calculate Cohen’s *d* effect sizes for AD and MCI patients vs controls (figure 1A). RVI-AD is a scalar correlation coefficient between region-wise *z* values for the individual and the AD-control effect sizes. Higher RVI values in SZ patients would imply a higher similarity of the white matter regional deficit patterns in SZ toward that of AD. RVI-SZ was calculated using effect sizes from the ENIGMA sample.^39^

#### Hypothesis Testing

Effect sizes of white matter FA in AD and MCI calculated from the ADNI sample were used to derive RVI-AD and RVI-MCI in SZ patients and controls. Hypothesis testing was performed in 3 steps: (1) Testing if RVI-AD is elevated in SZ patients in the discovery sample, followed by confirmatory testing in 2 replication cohorts and age-group analysis; specificity testing was performed using RVI-MCI values. (2) Testing the reverse association where RVI-SZ based on the ENIGMA SZ pattern is significantly elevated in AD patients; specificity testing was performed in MCI patients. (3) Testing that RVI-AD is associated with cognitive deficits in AD patients and SZ patients.

The age-group analysis was tested by dividing the discovery and replication cohorts into 4 age groups: 18–29, 30–39, 40–49, and 50–65 (supplementary table S5) and evaluating patient-control differences within each age category.

The associations between RVI and cognition were tested using a linear model:

$$\begin{array}{ll} & Cognition\;∼\;A+\;\beta_{sz}\cdot DX_{sz}or\;\beta_{AD}\cdot DX_{AD}\\ & +\beta_{RVI-AD}\cdot RVI-ADor\;\beta_{RVI-SZ}\cdot RVI-SZ \end{array}$$

where “cognition” stands for the cognitive measures and DX for diagnosis (coded as follows: in the SZ sample: SZ = 1, control = 0; in the ADNI sample: AD = 1, control = 0). Collinearity was examined using the variance inflation factor (<5.0 is deemed acceptable). Effects of symptoms, antipsychotic medication dose, and smoking status (modeled as current smokers or not) were similarly explored in regression analyses. Group comparisons of RVI values were performed by ANOVA. All statistical significance thresholds were corrected for multiple comparisons by false discovery rates (FDR).

## Results

### Patterns of Regional White Matter Deficits in AD, MCI, and SZ

Five samples were analyzed to test the above hypotheses—the ADNI samples^40^ for AD and MCI and 3 SZ samples—a discovery sample from Baltimore, MD; a replication sample from Beijing, China; and a replication sample from the worldwide meta-sample from ENIGMA, which is the largest white matter imaging sample in SZ.^41^ Each sample had its own healthy controls. Details on sample size, demographic (supplementary tables S1 and S2), and imaging (supplementary table S3) information are in the Methods section and supplementary information. White matter microstructures were measured by FA of DTI. The ranks of regional FA effect sizes between patients and controls for each cohort are shown in figure 1 and supplementary table S4. In AD, the largest effect sizes were in the fornix (FX) followed by the splenium of the corpus callosum (SCC) (figure 1A). In MCI, the largest effect sizes were in the SCC and FX (figure 1B). In the 3 SZ cohorts, patients showed the largest effect sizes in the anterior corona radiata (ACR), anterior limb of the internal capsule (ALIC), FX, and body of the corpus callosum (BCC) (figures 1C–E). The regional patient-control effect sizes in the discovery and replication cohorts were significantly correlated with those reported by ENIGMA (*r* = .94 and .85, *P* < .01, for discovery and replication cohorts, respectively).

### Elevated White Matter RVI-AD in SZ in the Discovery Sample

**Fig. 2.**
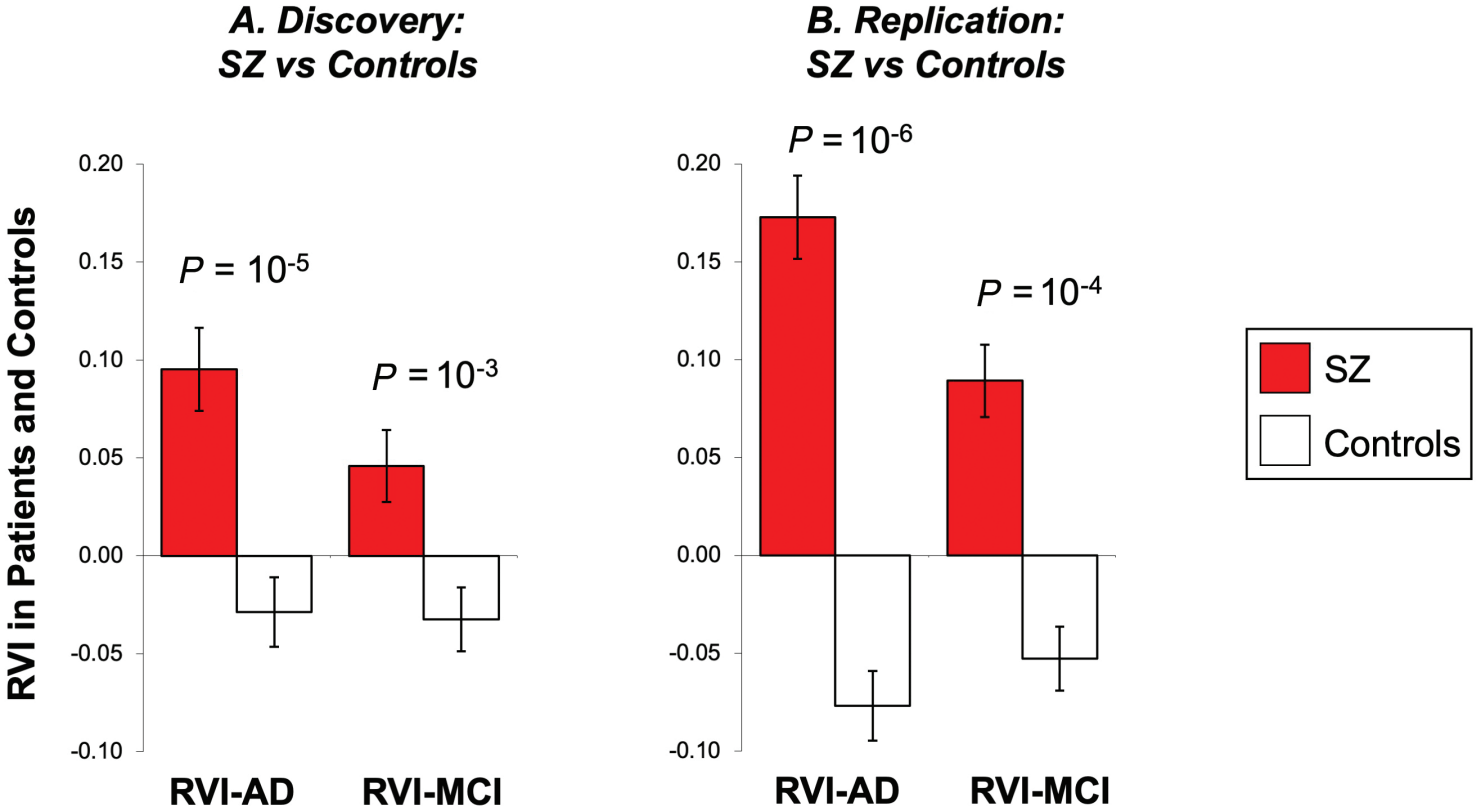
Patient-control differences in the average RVI-AD and RVI-MCI values for the discovery and replication cohorts. *Note*: RVI, regional vulnerability index; AD, Alzheimer’s disease; MCI, mild cognitive impairment.

**Fig. 3.**
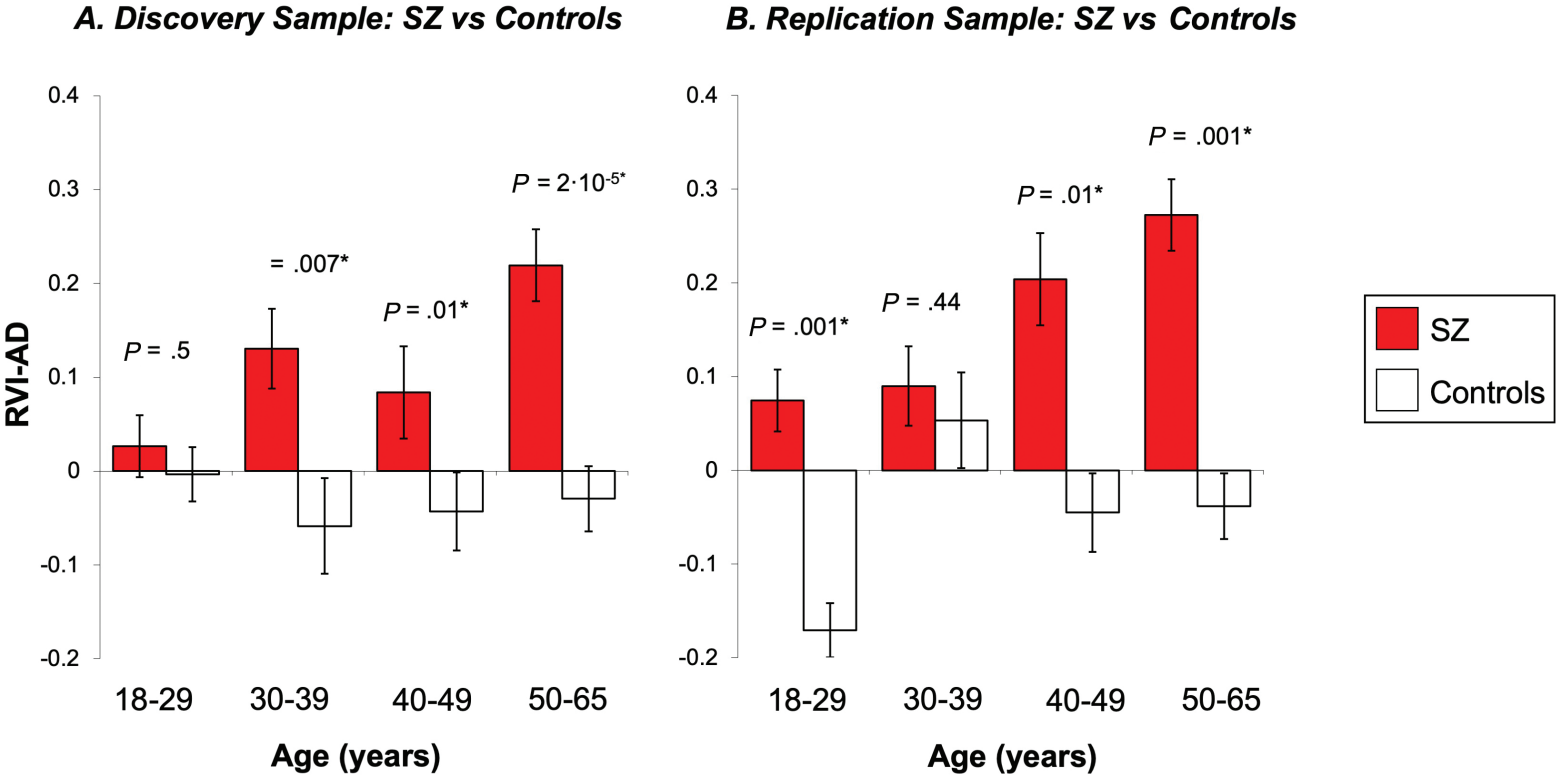
Significant age-related increases of RVI-AD values for schizophrenia (SZ) (**A** and **B**). *Significant after false discovery rates (FDR) correction for multiple comparisons. *Note:* RVI, regional vulnerability index; AD, Alzheimer’s disease.

SZ patients had significantly higher RVI-AD than controls (Cohen’s *d* = 0.44, *t* = 4.5, *P* = 1·10^–5^) figure 2A). RVI-AD showed significant and positive Spearman correlations with age in SZ patients (*r* = .21, *P* < .001) but not in controls (*r* = −.03, *P* = .8). SZ patients had significantly higher RVI-AD compared with controls starting at age 30 (figure 3A).

### Elevated White Matter RVI-AD in SZ in the Replication Sample

SZ patients again showed significantly higher RVI-AD than controls (Cohen’s *d* = 0.78, *t* = 5.1, *P* = 9·10^–7^) (figure 2B). There were no significant differences between the discovery and replication cohorts in RVI-AD (*P* = .07) (compare figure 2A vs 2B). SZ patients had significantly higher RVI-AD compared with controls starting at age 40 (figure 3A). The significant correlations of RVI-AD with age were also replicated in SZ patients (*r* = .29, *P* < .001) (figure 3B). The correlation between RVI-AD and age was not significant in controls (*r* = .18, *P* = .2).

### Replication of Elevated White Matter RVI-AD in SZ Using ENIGMA Sample

**Fig. 4.**
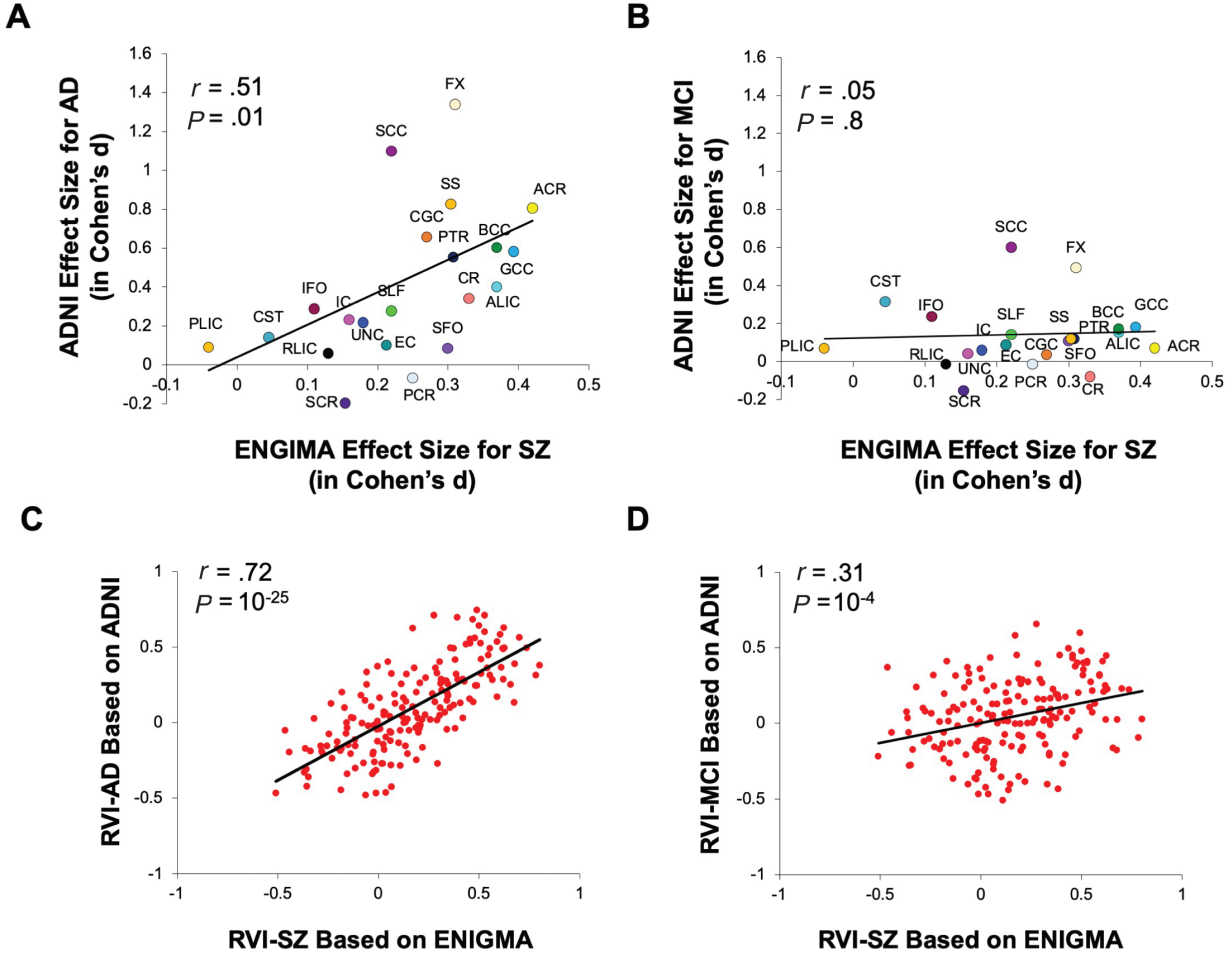
On white matter tract level (**A** and **B**): **A**. Significant correlation of regional deficit (in Cohen’s *d* values) patterns across the 22 major white matter tracts for Alzheimer’s disease (AD; calculated from ADNI) and schizophrenia (SZ; calculated from ENIGMA). **B**. No such correlation was observed between MCI and SZ. Similarly, on individual SZ patient level (**C** and **D**): **C**. regional vulnerability index (RVI) for AD (calculated from ADNI) and RVI for SZ (calculated from ENIGMA) showed a robust correlation in SZ patients (from the discovery sample, same below); **D**: correlation between RVI for MCI and RVI for SZ in SZ patients. *Note*: MCI, mild cognitive impairment.

The ENIGMA SZ pattern of regional white matter deficits was significantly correlated with the pattern for white matter deficits in AD (*r* = .55, *P* = .01) (figure 4A). The RVI-AD (calculated from ADNI) and RVI-SZ (calculated from ENIGMA) in SZ patients were highly correlated (*r* = .65, *P* = 10^–25^) (figure 4C). Therefore, despite the very different age ranges, etiologies, and neuropathologies, the white matter deficit patterns in SZ patients showed significantly elevated similarity to white matter deficit patterns observed in AD patients across the samples.

### Relative Specificity Testing by Comparing With MCI

SZ patients also showed elevated RVI-MCI in both the discovery (*t* = 3.3, *P* = .002) and replication sample (*t* = 3.8, *P* = 2·10^–4^) (figure 2B). The ENIGMA SZ pattern of white matter deficits was not significantly correlated with those in MCI (*r* = .10, *P* = .6) (figure 4B). The correlation between RVI-MCI and RVI-SZ was also significant (*r* = .31, *P* = 10^–4^) (figure 4D) but the *r* value was significantly smaller than the correlation between RVI-AD and RVI-SZ (*z* test of the correlation coefficients = 3.5, *P* = 4·10^–4^).

### Testing the Reverse Association: Elevated RVI-SZ in AD

RVI-SZ values were derived from the ENIGMA SZ sample and used to calculate RVI-SZ in AD and MCI patients and controls in ADNI. RVI-SZ was significantly higher in AD patients compared with MCI patients (*P* = .009) and normal controls (*P* = 8·10^–5^) (average RVI-SZ = 0.15 ± 0.04, 0.00 ± 0.3, and −0.06 ± 0.4 for AD, MCI, and controls, respectively). MCI patients showed no significant difference in average RVI-SZ values compared with controls (*P* = .21).

### RVI-AD on Cognition and Clinical Characteristics

Processing speed has been considered the cognitive measure most robustly associated with schizophrenia^42,43^ and schizophrenia-related white matter deficits.^9,44^ We found that RVI-AD significantly correlated with processing speed in the discovery (*r* = −.34, *P* = 1·10^–4^) and replication samples (*r* = −.27, *P* = 2·10^–4^). One of the standard tests for cognitive deficits for AD is the ADAS-Cog.^45^ RVI-AD was significantly and positively correlated with the ADAS-Cog in the full ADNI sample (*r* = .42, *P* = 10^-10^). Interestingly, RVI-SZ was also significantly correlated with ADAS-Cog in the full ADNI sample (*r* = .33, *P* = 1·10^–6^). Finally, psychiatric symptoms were measured in the discovery and replication samples using the Brief Psychiatric Rating Scale (BPRS). No significant associations between RVI-AD and BPRS total or any subscale scores were detected (all *r* < .1, *P* > .20). There were also no significant effects of current antipsychotic medication dosage or current smoking status (all *P* > .15) on the RVI-AD scores in the discovery or replication datasets.

## Discussion

Cognitive enhancing compounds have been tested as prospective frontline treatments for both SZ and AD but the effort has thus far has met with costly failure.^46^ Identifying the overlapping pathophysiological mechanisms for cognitive deficits in SZ and AD may guide and focus research on effective treatments that target the shared deficits between them. We observed a striking similarity between white matter deficit patterns in SZ and AD that are both replicable and associated with core cognitive deficits in the respective illnesses. The RVI-AD values were positively correlated with age in patients but not in controls. Patients started to show significantly higher RVI-AD in the fourth decade of life in the discovery cohort and during the fifth decade in the replication cohort. In all 3 datasets (discovery, replication, and ADNI), higher RVI-AD values were associated with worse neurocognitive performance. Our findings propose bringing white matter-related biomarkers to the forefront of efforts to identify treatment targets for cognitive deficits in both illnesses.

The significant correlation of white matter patient-control regional effect size patterns between SZ and AD was driven by a significant reduction in the integrity of the associative white matter tracts, including the ACR, FX, and commissural fibers in the genu of corpus callosum (GCC) and BCC while relatively sparing the motor and sensory fibers such as the corticospinal tract and posterior limb of the internal capsule in both SZ and AD (figure 1). ACR, GCC, and BCC contain the primary fibers connecting the ipsilateral and interhemispheric prefrontal cortex and are critical for higher cognitive functions. Among all brain regions, the frontal white matter volume had the largest evolutionary increase in humans compared with other species,^47^ implying that the regions showing the strongest impairment in both SZ and AD (figure 4C) are also the phylogenetically most-expanded brain tissue in humans. The FX is another associative fiber with substantial impairment in both SZ and AD. The FX fiber-tract links the hippocampus with the lateral hypothalamus and frontal cortical regions. Hippocampal shrinkage is a hallmark of AD,^48,49^ and hippocampal volume in SZ shows the largest reduction among all subcortical regions.^50^ These neurobiological signatures common to SZ and AD may explain their associations with similar regional deficit patterns captured by the RVI approach.

RVI is a simple correlation between an individual and the expected deficit pattern derived from a representative disease sample.^32^ The RVI is calculated after correcting the regional traits for age and sex. Therefore, the aging-related increases in RVI-AD in patients support the heuristics of SZ as a progressive neurological disease with an elevated risk of dementia.^51–58^ These heuristics are further supported by evidence for *the accelerated decline of the associative white matter tracts in patients with SZ.*^30,59,60^*Thus, the higher white matter RVI-AD in SZ patients is likely associated with detrimental interactions between SZ risk factors and aging of cerebral white matter.* RVI does not measure the underlying neurobiological mechanisms. However, because RVI quantifies the similarity of the anatomic deficit pattern to the representative disease pattern, it can capture shared brain deficit patterns between different diseases and allow for the inference of intersecting anatomic mechanisms.

The hypothesis of similarity in the underlying mechanisms for the shared white matter deficits between AD and SZ is still a subject to debate. Contemporary medicine has largely dismissed Kraepelin’s definition of SZ as premature dementia^61^ due to the lack of evidence for neurodegenerative cognitive decline in most SZ patients.^6^ The lack of amyloid-β and τ-protein pathologies in most SZ postmortem studies further suggests nonoverlapping pathophysiology.^2,3^ Despite these clear etiopathological differences, neuroimaging studies have shown similarities in deficits patterns between AD and SZ. For example, anatomical integrity in the temporal lobe and hippocampal regions are highly impacted in both illness,^62^ yet the field lacks robust comparative studies to determine if the 2 illnesses intersect as Kraepelin once delineated with conviction. This question is revived by the evidence of a higher risk for developing dementia in SZ.^1^ Using modern imaging methods, our findings provide tantalizing evidence for a relationship between SZ and AD at the white matter level.

In AD, white matter deficits can be secondary to neuronal amyloid-β and amyloid precursor protein accumulation^63^ in the frontal and temporal lobes that eventually impact the associative tracts.^64,65^ In SZ, white mater deficits may be due to the abnormal expression of oligodendrocyte lineage genes or oligodendrocyte cellular dysfunctions.^66,67^ The reciprocal rise in RVI-SZ in AD and RVI-AD in SZ may reflect different underlying pathology that converges on similar white matter structures as the disorders progress.

Conversely, there are arguments for the sharing of etiological mechanisms between 2 disorders. For example, the frontal white matter has high metabolic demands and is vulnerable to cardiovascular risks.^68,69^ SZ patients have a shorter lifespan, and the leading cause of their premature mortality is cardiovascular diseases.^70^ In parallel, vascular-related white matter damage predicts the development of AD, leading to the emerging 2-hit theory on AD etiology, whereby vascular insults constitute the first hit while the accumulation of amyloid-β is the second.^71,72^ Therefore, cardiovascular risks may present a common etiology to explain the shared white matter deficit patterns.

Another question is whether the overlapping white matter regional deficit patterns in AD and SZ are related to general cognitive deficits and, therefore, nonspecific. However, the correlation between regional deficit patterns in SZ and MCI was not significant (figure 4B). Individuals with MCI have an unfavorable aging trajectory that leads to deficits in the FX,^73,74^ similar to AD and SZ. However, MCI patients showed minimal deficits in the ACR, GCC, BCC, and other associative tracts. The specificity testing suggests that the sharing of deficit patterns between SZ and AD is not simply due to age-related cognitive decline as in MCI.

We also observed that RVI-AD was positively correlated with age in SZ (figures 3A and 3B), such that the similarity to AD in SZ patients became stronger with age. This finding is consistent with recent “brain age” analyses showing that SZ had the largest accelerated aging rate among all psychiatric illnesses (Cohen’s *d* = 0.54), and AD has an even larger effect size (*d* = 1.03).^75^ This may argue for the sharing of an age-related underlying pathology for the 2 otherwise distinct illnesses.

Our study is limited by the lack of longitudinal data to confirm if the RVI-AD score would track the risk for dementia and cognitive decline in SZ patients. Only FA was used to index white matter abnormalities and other diffusion parameters were not explored. We chose FA as it shows a higher sensitivity to SZ deficits compared with other parameters.^39^ Additionally, the testing of RVI-cognition associations was limited by differences in the cognitive tests administered across the discovery and replication samples, especially the ADNI sample where the cognitive tests were designed for the diagnosis of dementia and, therefore, may suffer from score compression in non-AD subjects due to ceiling effects. Lastly, a potential explanation for the similarity of WM deficits in SZ and AD is the use of antipsychotic medications in both disorders. However, we consider this to be unlikely due to (1) the lack of association between RVI and medication dose and (2) the elevation of RVI-SZ values at the treatment initiation stage.^32^ The latter suggests that white matter deficits are already established in patients prior to the treatment onset.

A shared aim in SZ and AD research is to discover therapeutic interventions that can restore, prevent, or slow down the progression of cognitive deficits in these diseases. We demonstrated an unambiguous overlap in white matter deficit patterns between SZ and AD. This pattern was associated with the disease-related cognitive deficits and appeared to be disease-specific. Development of future treatments and therapies to prevent and treat cognitive deficits in SZ and AD should consider targeting white matter-related mechanisms.

## Supplementary Material

sbaa078_suppl_Supplement_Material
