# Supplementary material for: A White Matter Connection of Schizophrenia and Alzheimer’s Disease
Source: Schizophr Bull. 2020 Jul 18;47(1):197–206. doi: 10.1093/schbul/sbaa078 (PMC7825012; doi:10.1093/schbul/sbaa078)
Supplement: sbaa078_suppl_Supplement_Material [file sbaa078_suppl_supplement_material.docx]

**Supplementary Information (SI)**

**Additional Clinical Information and Imaging Methods**

ADNI

ADNI2 baseline DTI, demographics, and diagnosis data were downloaded from the ADNI database (<http://adni.loni.usc.edu>). Clinical information is in **Table S1**. Further details about ADNI2 inclusion and exclusion criteria can be found here: https://www.nia.nih.gov/alzheimers/clinical-trials/alzheimers-disease-neuroimaging-initiative-2-adni2.

Imaging data was collected using GE Medical Systems 3 Tesla MRI scanners equipped with multichannel coils. Data was collected using a spin-echo, EPI sequence with isotropic spatial resolution of 2.7 × 2.7 × 2.7 mm. The sequence parameters were TE/TR = 68/9000 ms, FOV=256mm, axial slice orientation with 50 slices and no gaps, 41 isotropically distributed diffusion weighted directions, two diffusion weighting values (*b*=0 and 1000 s/mm^2^) and five *b*=0 images (**Table S4**).

Discovery Sample

The discovery sample consisted of mega-analytical aggregation of three cohorts collected at the Maryland Psychiatric Research Center. The local Internal Review Boards approved the studies, and informed written consent was obtained from all participants. Uniform cognitive and clinical assessment and exclusion criteria were maintained across the three cohorts. SZ patients were diagnosed with either schizophrenia or schizoaffective disorder as determined by the Structured Clinical Interview for DSM-IV or IV-TR (SCID). Each cohort contained its own healthy control group. All controls had no Axis I psychiatric disorder as determined by the SCID. With the exception of nicotine, all participants were excluded if they had DSM-IV substance abuse in the last 3 months or substance dependence within the past 6 months. Other exclusion criteria included diagnosis with uncontrolled hypertension, type 2 diabetes, heart disorders, or a major neurological event such as stroke or transient ischemic attack. Further clinical information is in **Table S2**.

Cohort A

Imaging data was collected using a Siemens 3T TRIO MRI (Erlangen, Germany), running VB17 software and equipped with a 32-channel RF head coil. Data was collected using a spin-echo, EPI sequence with a spatial resolution of 1.7×1.7×3.0mm. The sequence parameters were: TE/TR=87/8000ms, FOV=200mm, axial slice orientation with 50 slices and no gaps, 64 isotropically distributed diffusion weighted directions, two diffusion weighting values (*b*=0 and 700 s/mm^2^) and five *b*=0 images (**Table S4**)

Cohort B

Imaging data was collected using a Siemens 3T TRIO MRI running VB13 software and equipped with a 12-channel RF head coil. Data was collected using a spin-echo, EPI sequence with a spatial resolution of 1.8×1.8×3.0mm. Sequence parameters were: TE/TR=92/6700ms, FOV=210mm, axial slice orientation with 49 slices and no gaps, 30 isotropically distributed diffusion weighted directions, two diffusion weighting values (*b*=0 and 1000 s/mm^2^) and three *b*=0 values (**Table S4**).

Cohort C

Imaging data was collected using a Siemens 3T Allegra MRI running VA19 software and using a spin-echo, EPI sequence with a spatial resolution of 1.7×1.7×4.0mm. The sequence parameters were: TE/TR=87/5000ms, FOV=200mm, axial slice orientation with 35 slices and no gaps, 12 isotropically distributed diffusion weighted directions, and two diffusion weighting values (*b*=0 and 1000 s/mm^2^). The entire protocol was repeated five times to improve signal-to-noise ratio (**Table S4**)

Replication Cohort

The patients were recruited from Beijing Huilongguan Hospital, China. Controls were recruited through local advertisement. All patients met DSM-IV criteria for SZ. Participants had a homogeneous Chinese background. All participants provided written informed consent according to the Helsinki Declaration. Additional clinical information is in **Table S2**. Imaging data was collected using a 3T Prisma MRI scanner (Erlangen, Germany) at the Imaging Research Center of the Beijing Huilongguan Hospital, equipped with a 64-channel RF head coil. DTI data was collected using a spin-echo, EPI sequence with a spatial resolution of 1.7×1.7×1.7 mm. The sequence parameters were: TE/TR=87/8000 ms, FOV=200 mm, axial slice orientation with 82 slices and no gaps, 98 isotropically distributed diffusion-weighted directions, two diffusion weighting values (*b*=0 and 1000 s/mm^2^) and five *b*=0 images (**Table S4**).

**Supplementary Tables**

**Table S1**. Demographic information and clinical characteristics of the ADNI sample

|  | ADNI Cohort | | | |
| --- | --- | --- | --- | --- |
|  | AD (29M/19F) | MCI (73M/44F) | Controls (24M/29F) | p value |
| Age (mean±sd) | 74.9±8.7 | 72.0±7.7 | 72.4±6.0 | 0.8 |
| Range of Age (years) | 56-90 | 50-90 | 60-90 | n/a |
| Age-of-Onset (years) | 73.4±8.1 | n/a | n/a | n/a |
| Illness Duration (years) | 1.6±2.0 | n/a | n/a | n/a |
| Education (years) | 15.5±2.9 | 15.8±2.7 | 16.6±2.8 | 0.9 |
| Ethnicity (CEU/AA/Other) | 42/1/5 | 98/3/16 | 44/2/7 | 0.9 |
| Mini-Mental Status Examination | 23.3±1.9 | 27.9±1.6 | 28.8±1.4 | 0.006 |
| Clinical Dementia Rating Scale | 4.7±1.5 | 1.3±0.7 | 0.3±0.1 | 0.002 |
| Alzheimer’s Disease Assessment Scale | 29.6±9.4 | 16.3±7.0 | 8.7±4.3 | 0.008 |

**Table S2**. Demographic information for the discovery and replication SZ cohorts

|  | Discovery Cohort  (173 patients / 230 controls) | | | | | | | | | Replication Cohort  (122 patients / 64 controls) | | |
| --- | --- | --- | --- | --- | --- | --- | --- | --- | --- | --- | --- | --- |
|  | Cohort A | | | Cohort B | | | Cohort C | | |  |  |  |
|  | Patients (44M/21F) | Controls (61M/79F) | p value | Patients (31M/15F) | Controls (35M/17F) | p value | Patients (47M/15F) | Controls (23M/15F) | p value | Patients (57M/65F) | Controls (38M/26F) | p value |
| Age (mean±sd) | 34.6±11.7 | 35.6±13.8 | 0.61 | 35.3±9.5 | 40.3±11.7 | 0.006 | 37.7±12.8 | 39.1±13.6 | 0.63 | 41.1±13.2 | 39.1±13.3 | 0.35 |
| Range of Age | 18-65 | 18-65 | n/a | 20-60 | 20-60 | n/a | 18-61 | 18-61 | n/a | 18-65 | 18-65 | n/a |
| BPRS/PANSS Total | 30.7±10.7 | n/a | n/a | 30.7±9.6 | n/a | n/a | 31.2±8.6 | n/a | n/a | 74.1±13.6 | n/a | n/a |
| Age-of-Onset (years) | 18.1±8.5 | n/a | n/a | 18.9±7.8 | n/a | n/a | 18.5±7.5 | n/a | n/a | 24.6±8.8 | n/a | n/a |
| Illness Duration (years) | 21.5±15.1 | n/a | n/a | 20.1±13.7 | n/a | n/a | 19.7±13.6 | n/a | n/a | 21.4±12.7 | n/a | n/a |
| Education (year) | 13.0±2.0 | 14.0±2.5 | 0.003 | 13.0±2.2 | 14.5±2.5 | 0.001 | 12.2±2.2 | 14.9±2.5 | 0.001 | 12.1±3.1 | 13.2±2.6 | 0.01 |
| Ethnicity (CA/AA/Other) | (29/27/2) | (89/31/3) | 0.1 | (23/23/0) | (29/23/0) | 0.41 | (28/24/0) | (19/8/1) | 0.32 | 122 Han Chinese | 64 Han Chinese | 1.0 |
| Medication Dose (CPZ) | 400±352 | n/a | n/a | 630±544 | n/a | n/a | 454±372 | n/a | n/a | 454±395 | n/a | n/a |
| Current Smokers | 29% | 27% | 0.95 | 55% | 35% | 0.15 | 68% | 44% | 0.01 | 35% | 29% | 0.4 |
| Processing Speed | 55.2±15.7 | 73.5±15.6 | 1.2·10^-10^ | 47.3±11.3 | 64.9±11.6 | 1.0·10^-9^ | 51.6±14.9 | 68.9±14.6 | 1.3·10^-7^ | n/c | n/c | n/c |
| Working Memory | 15.9±5.5 | 20.1±4.3 | 1.0·10^-8^ | 17.0±4.5 | 21.2±4.0 | 1.5·10^-7^ | 17.5±4.8 | 21.1±3.8 | 2.0·10^-4^ | n/c | n/c | n/c |

Psychiatric symptoms were ascertained used Brief Psychiatric Rating Scale (BPRS) in discovery and Positive and Negative Symptoms Scale (PANSS) in the replication cohorts. Medication dose was coded as chlorpromazine equivalent (CPZ mg). Processing speed and working memory were ascertained using Digit Symbol Coding task of the WAIS-3[1] and Digit Sequencing Test[2], respectively. CA: Caucasian American. AA: African American.

**Table S3**. Diffusion tensor imaging parameter information for the ADNI, SZ and MDD samples.

| Cohort | Scanner | TE (ms) | TR (ms) | In-plane resolution (mm) | N slices/Slice Thickness (mm) | Diffusion b-value (s/mm^2^) | N-gradients | N-b0 | Duration (min) |
| --- | --- | --- | --- | --- | --- | --- | --- | --- | --- |
| ADNI cohort | GE 3T | 68 | 9000 | 2.7x2.7 | 50/2.7 | 1000 | 41 | 5 | 8 |
| Discovery: Sample A | Siemens 3T Allegro | 87 | 8000 | 1.7x1.7 | 50/3.0 | 700 | 64 | 5 | 8 |
| Discovery: Sample B | Siemens 3T Trio | 92 | 6700 | 1.8x1.8 | 49/3.0 | 1000 | 30 | 3 | 7 |
| Discovery: Sample C | Siemens 3T Trio | 85 | 5000 | 1.7x1.7 | 35/4.0 | 1000 | 12 | 5 | 10 |
| Replication | Siemens 3T Prisma | 87 | 8000 | 1.7x1.7 | 82/1.7 | 1000 | 98 | 5 | 8 |

**Table S4**. Whole-brain average and regional FA effect sizes (controls > patients) for six cohorts ascertained in this study.

|  | AD Cohen's d-value  (p-value) | MCI Cohen's d-value  (p-value) | Discovery-SZ Cohen’s d-value (p-value) | Replication-SZ Cohen’s d-value (p-value) | ENIGMA-SZ Cohen’s d-values ^#^ |
| --- | --- | --- | --- | --- | --- |
| Whole-brain average | 0.99 ( 2·10^-5^)* | 0.30 (0.10) | 0.63 (2·10^-9^)* | 0.59 (2·10^-6^)* | 0.42 (5·10^-24^)* |
| Anterior Corona Radiata (ACR) | 0.80 (4·10^-4^)* | 0.07 (0.7) | 0.71 (2·10^-11^)* | 0.42 (2·10^-3^)* | 0.40 (9·10^-19^)* |
| Anterior Limb of Internal Capsule (ALIC) | 0.40 (0.07) | 0.15 (0.4) | 0.64 (1·10^-9^)* | 0.45 (1·10^-3^)* | 0.37 (2·10^-15^)* |
| Body of Corpus Callosum (BCC) | 0.81 (5·10^-4^)* | 0.32 (0.07) | 0.53 (4·10^-7^)* | 0.49 (5·10^-4^)* | 0.40 (3·10^-18^)* |
| Cingulum (CGC) | 0.66 (4·10^-3^)* | 0.03 ( 0.8) | 0.36 (4·10^-4^)* | 0.46 (1·10^-3^)* | 0.27 (3·10^-9^)* |
| Corona Radiata (CR) | 0.24 0.3) | -0.08 ( 0.7) | 0.61 (6·10^-9^)* | 0.33 ( 0.02) | 0.33 (3·10^-17^)* |
| Cortico-Spinal Tract (CST) | 0.45 (0.04) | 0.31 (0.08) | 0.28 (7·10^-3^) | 0.03 ( 0.9) | 0.05 (0.24) |
| Corpus Callosum (CC) | 0.81 (3·10^-4^) | 0.32 (0.08) | 0.45 (2·10^-5^)* | 0.45 (1·10^-3^)* | 0.40 (8·10^-19^)* |
| External Capsule (EC) | 0.10 (0.7) | 0.09 (0.6) | 0.48 (4·10^-6^)* | 0.41 (5·10^-3^)* | 0.21 (1·10^-7^)* |
| Fornix (FX) | 1.34 (4·10^-8^)* | 0.49 (6·10^-3^)* | 0.58 (3·10^-8^)* | 0.50 (2·10^-7^)* | 0.31 (7·10^-12^)* |
| Genu of Corpus Callosum (GCC) | 0.60 (8·10^-3^)* | 0.17 ( 0.33) | 0.68 (1·10^-10^)* | 0.36 ( 0.01) | 0.37 (1·10^-18^)* |
| Internal Capsule (IC) | 0.22 (0.3) | 0.06 ( 0.7) | 0.46 (1·10^-5^)* | 0.16 ( 0.3) | 0.18 (2·10^-5^)* |
| Inferior Frontal Occipital fasciculus (IFO) | 0.29 (0.2) | 0.24 (0.2) | 0.21 ( 0.04) | 0.12 ( 0.4) | 0.11 (3·10^-3^)* |
| Posterior Corona Radiata (PCR) | -0.07 (0.8) | -0.02 ( 0.9) | 0.48 (4·10^-6^)* | 0.17 ( 0.2) | 0.25 (2·10^-12^)* |
| Posterior Limb of Internal Capsule (PLIC) | 0.09 (0.7) | 0.07 ( 0.0) | -0.02 ( 0.4) | -0.17 ( 0.2) | -0.04 (0.4) |
| Posterior Thalamic Radiation (PTR) | 0.55 ( 0.01)* | 0.12 ( 0.0) | 0.40 (1·10^-4^)* | 0.37 ( 0.01) | 0.31 (2·10^-18^)* |
| Retrolenticular Limb of the Internal Capsule (RLIC) | 0.06 (0.80) | -0.02 ( 0.2) | 0.11 ( 0.2) | 0.16 ( 0.2) | 0.13 (2·10^-3^)* |
| Splenium of Corpus Callosum (SCC) | 1.10 (4·10^-6^)* | 0.60 (9·10^-4^)* | 0.47 (5·10^-6^)* | 0.28 ( 0.04) | 0.22 (4·10^-6^)* |
| Superior Corona Radiata (SCR) | -0.20 (0.4) | -0.15 (0.4) | 0.35 (7·10^-4^)* | 0.14 ( 0.3) | 0.15 (8·10^-3^)* |
| Superior Fronto-Occipital Fasciculus (SFO) | 0.08 (0.7) | 0.11 (0.5) | 0.57 (5·10^-8^)* | 0.39 (7·10^-3^)* | 0.30 (4·10^-8^)* |
| Superior Longetudinal Fasciculus (SLF) | 0.27 (0.2) | 0.14 (0.4) | 0.49 (2·10^-6^)* | 0.23 ( 0.1) | 0.22 (6·10^-8^)* |
| Sagittal Striatum (SS) | 0.82 (4·10^-4^)* | 0.12 ( 0.5) | 0.58 (4·10^-8^)* | 0.36 ( 0.01) | 0.30 (5·10^-14^)* |
| Uncinate Fasciculus (UNC) | 0.23 (0.3) | 0.04 (0.8) | 0.28 (6·10^-3^) | 0.23 ( 0.10) | 0.16 (9·10^-6^)* |

^#^ ENIGMA effect sizes are taken from[3]. * Statistically significant after false discovery rates (FDR) correction for multiple comparisons

**Table S5.** Sample composition for four age groups in discovery and replication cohorts.

|  | Discovery Cohort | | Replication Cohort | |
| --- | --- | --- | --- | --- |
| Age Groups | Patients | Controls |  |  |
| *Age 18-29* |  |  |  |  |
| N (M/F) | 72 (54/18) | 84 (45/39) | 33 (15/18) | 25 (18/7) |
| Age | 23.5±3.5 | 23.1±3.4 | 23.4±3.8 | 25.0±2.9 |
| *Age 30-39* |  |  |  |  |
| N (M/F) | 28 (22/6) | 32 (19/13) | 21(12/9) | 8(4/4) |
| Age | 34.3±3.1 | 34.0±3.3 | 34.2±2.3 | 33.8±2.7 |
| *Age 40-49* |  |  |  |  |
| N (M/F) | 40 (22/18) | 54 (26/28) | 27 (15/12) | 10 (5/5) |
| Age | 44.6±2.7 | 45.1±3.2 | 45.6±2.5 | 45.6±2.8 |
| *Age 50-65* |  |  |  |  |
| N (M/F) | 33 (24/9) | 60 (29/31) | 41 (15/26) | 21 (11/10) |
| Age | 54.8±3.8 | 55.6±3.8 | 54.6±3.5 | 54.8±3.1 |

References

1. Weshsler, D., *Wechsler Adult Intelligence Scale* 3rd edn. ed. 1997, San Antonio, TX: Psychological Corporation.

2. Keefe, R.S., et al., *The Brief Assessment of Cognition in Schizophrenia: reliability, sensitivity, and comparison with a standard neurocognitive battery.* Schizophrenia research, 2004. **68**(2-3): p. 283-97.

3. Kelly, S., et al., *Widespread white matter microstructural differences in schizophrenia across 4,375 individuals: results from the ENIGMA Schizophrenia DTI Working Group.* Mol Psychiatry, 2017. **23**(5): p. 1261-69.
